# The role of imaginary companion in the life of only children: a qualitative study

**DOI:** 10.1186/s12888-023-05360-0

**Published:** 2023-11-15

**Authors:** Zahra Yazdi Panah, Hassan Zareei Mahmoodabadi, Fahimeh Dehghani

**Affiliations:** Department of Psychology and Educational Sciences, Yazd University, Yazd, Iran

**Keywords:** Only child, Children, Imaginary companion, Qualitative content analysis, Mashhad city

## Abstract

**Introduction:**

An imaginary companion is an invisible or personified entity created by children for themselves. An imaginary companion typically serves as a companion to the child and plays a significant role in their life, especially for only children who may experience more loneliness compared to other children. This research was conducted to investigate the role of an imaginary companion in the lives of only children.

**Method:**

The present study was conducted using a qualitative method and a content analysis approach. Through purposeful sampling, a total of 34 preschool and primary school children, aged 6 to 9 years, from schools in Mashhad city, were selected until saturation was reached. They were subjected to semi-structured interviews. After data collection, the data were coded, and then the main and sub-themes were extracted.

**Results:**

The research findings were represented in the form of 196 statements, 28 sub-themes, and 9 main themes. The main themes included the role of an imaginary companion in alleviating loneliness, the role of an imaginary companion in amusement, the role of an imaginary companion in emotional regulation, conversations with an imaginary companion, guidance from an imaginary companion for good and bad behaviors, the assistance of an imaginary companion in tasks, helping to generate new scenarios, the advantages of having an imaginary companion, and the disadvantages of having one.

**Conclusion:**

Based on the aforementioned findings, it can be concluded that the presence of an imaginary companion can not only support children but also promote creativity and distance them from the virtual space and realities of the real world. Parental awareness of this matter can aid in the child’s growth, fulfill their needs, and, on the other hand, prevent potential harm to children.

## Introduction

Significant changes in the form and characteristics of families in Iran are evident [1]. The decline in birth rates has led to the transformation of extended families into nuclear families, and the desire for childbearing has decreased [2, 3]. This continuous decline has resulted in changes in family structure and an increase in single-child families [4], while parental interest in having a child is increasing. The rate of single-child families is reported to be around 7% in developing countries and over 15% in developed countries, with these figures rapidly increasing [5]. In Iran, due to cultural, social, and economic conditions, late marriage and divorce have contributed to the increase in single-child families [6].

Researchers believe that only children experience loneliness and maladjustment due to not having siblings [7]. However, subsequent studies have not confirmed these claims, and no significant differences have been observed in the personalities of only children compared to other children [8]. The feeling of loneliness and related themes, such as internalizing problems and fear of loneliness, are subjects that single adolescents have repeatedly referred to [6]. Sometimes, only children compensate for their loneliness by forming stronger relationships with themselves [9] or by creating a fantasy life that includes imaginary companions [10].

Bouldin and Pratt [11] define an imaginary companion as “a vividly imagined character that does not exist but is perceived as real by the child, with whom they play and refer to in conversation throughout the day.“ An imaginary companion can be completely invisible or take the form of a toy or object [12–16]. Imaginary companions can be human-like, but they can also take the form of other characters such as animals or abstract ideas [16, 17].

According to Vygotsky, cognitive development always results from the interaction of humans with each other, especially the interaction of an adult with a child [18]. According to Vygotsky, in pretend play, the child tries a variety of challenging activities and learns many new skills [18].Single children may be deprived of this game and thus turn to an imaginary friend. According to Vygotsky, self-centered or internal speech appears in a child between 3 and 7 years old. At this stage, children often talk to themselves in order to control their behavior. In fact, it is a natural thing in the process of child development [18].

Previous research has reported the prevalence of imaginary companions among children to be as high as 65%, although most studies indicate that between 20 and 35% of children have imaginary companions [19]. In one study, 28% of children aged 5 to 12 were found to have imaginary companions [20].

According to many studies, from single-child families to multi-child individuals, single-friend children experience loneliness, lack of childhood playmates and lack of siblings, and lack of support in youth and growing up [21].

Majors & Baines [22] believed that children with imaginary companions are likely to be first-born or only children. This suggests that children may use imaginary companions when others are not available to play with and rely on them to overcome moments of solitude. Even children who create imaginary companions and are not only children tend to have a greater age difference with their siblings compared to children without imaginary companions [22].

In the second half of the 20th century, the world experienced a demographic change. Fertility increased from five children per woman to 2.7 children worldwide [23]. However, new studies have shown that economic and psychological activity does not increase family size, because parents get the love they need from the presence of one or two children [24]. Shavazi [24] economic, social, political problems and people’s habit of having one or two children are among the factors of decreasing population growth.

Researchers have examined and identified several roles that imaginary companions may play in children’s lives [25, 26]. As children transition out of the preschool years and into early childhood, their parents may step back and give them more independence. Imaginary companions can fill this void and the absence of parents. Imaginary companions may play a role in improving self-esteem or even encouraging children to engage in more practical tasks like school assignments [27]. In the study by Hoff [28], imaginary companions of children served as a coach, a source of comfort, relieving boredom, and boosting self-esteem.

Studies have shown that having an imaginary companion in childhood has cognitive, social, and emotional benefits [29]. These studies have identified benefits such as a better understanding of different roles and pretending [30], improved reasoning and inference skills [28], better social interactions [29], increased use of private speech [31], and higher scores on measures of creativity and divergent thinking [25].

Considering the increase of single-child families in Iran and also the importance of siblings in the growth and development of children, examining the characteristics of single-child children is of particular importance. In Iran, there has not been a valid and organized scientific research with the theme of imaginary companions, and due to the importance of this issue in the lives of single children, this research was done.

This research was conducted with the aim of investigating the role of imaginary companions in the lives of single children. In this regard, this research seeks to answer the following questions:

1. What is the role of imaginary companions in the lives of single children?
2. What are the advantages and disadvantages of imaginary companions for only children?

## Research method

The present study was qualitative research. The method used in this research was qualitative content analysis. The research was conducted in a community of 6 to 9-year-old only children with imaginary companions in Mashhad, Iran. The age range of 6 to 9 years was selected based on the findings of Pearson et al. [20] for this study. The sampling method was purposive, the inclusion criteria were Parental consent form, individuals who were only children, had imaginary companions and fell within the specified age range were selected for interviews. The exclusion criteria was parents’ lack of consent, unwillingness to participate. Since the criterion for sample size was saturation, we reached saturation after conducting interviews with 30 individuals. However, to ensure robustness, interviews were conducted with an additional 4 individuals, resulting in a total of 34 interviews. This research was reviewed and approved at Yazd University with the ethics code IR.YAZD.REC.1401.054.

The procedure involved obtaining permission from the Ministry of Education and obtaining an ethics code. A participant was selected from the selected classes in schools, and after obtaining parental consent and ensuring the confidentiality of the interviews, the interviews were conducted. The interviews began with the question of whether they have a friend who can only be seen by them and with whom they interact. If the answer was positive (approximately 30 to 40% of the single-child students in each class had an imaginary friend), and their brief explanation matched the correct definition of an imaginary friend, the interview continued with further questions. The interviews with the children were recorded, and each interview lasted approximately twenty minutes to half an hour.

To ensure the accuracy and validity of the research data, the interviews were recorded and documented accurately. The entire process of conducting the research was described in detail. The results of the analysis were compared with the raw data to examine the consistency of the findings with the data. Additionally, the obtained results from data analysis were discussed and reviewed with experts to ensure validity and reliability.

After the interviews were meticulously coded, both primary and sub-themes were extracted. These steps were conducted in accordance with the guidelines provided by Prasad [32].

## Results

The average age of the research participants was 7.5 years. Out of the participants, 18 were girls and 16 were boys. Additionally, 22% of the fathers and 37% of the mothers of the only children had education levels higher than a bachelor’s degree.

This study aimed to investigate the role of imaginary companions in the lives of only children. The findings of the research were represented in the form of 196 statements, 28 sub-themes, and 9 main themes. According to the findings, the main roles of imaginary companions included: alleviating loneliness, engaging in play with the imaginary companion, the role of the imaginary companion in emotional regulation, conversing with the imaginary companion, guidance of the imaginary companion in distinguishing between good and bad behaviors, assisting the imaginary companion, and aiding in the absorption of new narratives. Additionally, the advantages and disadvantages of having an imaginary companion were also identified. The main themes will be further explained and described using the sub-themes (Table 1).

**Table 1:** Main and sub-themes regarding the role of imaginary companions in the lives of only children

Main Themes	Sub-Themes
Role of an imaginary companion in alleviating loneliness	Alleviating loneliness / Feeling less lonely during sleep / Substitute for siblings / Compensating for the lack of a real friend / Replacing the role of a mother
Role of imaginary companion in Amusement	Boredom / During watching movies / During playing / During leisure and entertainment activities
Role of imaginary companion in emotional regulation	Externalization, coping with stress, coping with anger, dealing with parental arguments and conflicts
Conversation with an imaginary companion	Talking to an imaginary companion / imaginary companion being a listener/ secret-keeping
Guidance from the imaginary companion for good and bad behavior	Encouragement for doing good behavior/ Encouragement for engaging in inappropriate behavior
Assistance from the imaginary companion in tasks	Assistance in daily tasks/ Academic Assistance
Assisting in adopting new schemes	Animation/personification
Advantages of an imaginary companion	Kindness/ Reduced screen time/ Control
disadvantages of an imaginary companion	Scaring/fostering attachment issues/controlling behavior

### Role of an imaginary companion in alleviating loneliness

Only children spend more time in solitude; they expressed this issue by phrases such as having an imaginary companion during moments of loneliness and avoiding loneliness through their presence. Participant number 23, a nine-year-old boy with working parents, admitted, “Once when I was very lonely, I saw a friend appear in front of me and said, ‘Don’t be alone. My friend looks like a beetle. When I’m alone, I don’t feel lonely. Once when I was very lonely, he said, ‘Loneliness is not a problem as long as I’m with you.‘” Furthermore, participant number 26, an eight-year-old boy with working parents, stated, “Because I’m alone, I make friends for myself. One is a sheep, and the other is a spider.“

Participant number 18, a six-year-old girl with working parents, expressed, “I feel a little lonely. My mom doesn’t give me siblings; she says I’ll have them when I grow up. So, I found this friend for myself when I was four years old, so I wouldn’t be alone. She has become like a sister to me, and we’re always together.“

### Role of imaginary companion in amusement

Only children create imaginary companions when they feel bored. Participant number 28, a nine-year-old boy with working parents, stated, “I spend time with my imaginary companion and have fun with them because there’s no one else, and I get bored.“ Additionally, only children create friendships while watching movies so that their imaginary companions can watch the movie with them, enjoy the film, and then engage in related play activities afterward. Participant number 4, a seven-year-old girl with a stay-at-home mother, said, “We watch movies together, and then we talk about the characters and the movies we’ve seen in real life, and we act them out.“

### Role of imaginary companion in emotional regulation

An imaginary companion helps only children regulate their emotions when facing life challenges. Children use their imaginary companions as a source of support when encountering stressful and fearful events. Participant number 20, a six-year-old boy, expressed, “One day we had an accident on a bridge, and I was very scared. Then he came, I call him Reza.“ Lastly, a sub-theme emerged during parental arguments and conflicts, where creating an imaginary companion during such times serves to empathize and connect with the child. Participant number 2, a seven-year-old girl with a stay-at-home mother, described it as follows: “When my mom and dad argue, I go to my room, cry, and talk to my friend. I’m afraid they won’t like each other anymore or that they won’t make up.“

### Conversation with an imaginary companion

Only children create an ideal companion by creating an imaginary friend with whom they can have ideal conversations. For example, participant number 15, a nine-year-old girl with a stay-at-home mother, said, “I talk to him, I tell him all the news, about my friends and the things I’ve done. I tell him the stories I know, and we talk a lot in general.“ Additionally, children trust their imaginary companions and confide their secrets to them. For instance, participant number 21, an eight-year-old boy with a stay-at-home mother, stated, “There are things that only a person knows, and nobody else knows. I tell them to my imaginary friend because I’m sure he won’t tell anyone since I’m the only one who can see him.“

### Guidance from the imaginary companion for good and bad behavior

Only children described the sub-theme of enjoining good behavior with phrases such as asking right and wrong questions and encouraging them to do the right thing. For example, participant number 22, a nine-year-old boy with working parents, admitted, “I ask them questions like I ask if the behavior my friend just did was right or not.“ Participant number 21, an eight-year-old boy with a stay-at-home mother, also stated, “I see him at home when I’m studying, and he says, ‘Study your lessons to become the top student.‘” The sub-theme of encouraging inappropriate behavior was also present, where children described tempting their imaginary companions to engage in wrong actions and encouraging them to do so. For instance, participant number 5, a seven-year-old girl with working parents, said, “Sometimes when my dad argues with me and raises his voice, he tells me to rebel against him and say all the bad things I want to say. But I say no because it would hurt my dad’s feelings.“

### Assistance from the imaginary companion in tasks

The sixth main theme discusses the role of imaginary companions as a helper and assistants in tasks. Only children described the sub-theme of help in daily tasks by using phrases such as “helping with tasks when tired.“ Participant 10, a nine-year-old girl with a stay-at-home mother, stated, “My imaginary friend came when I was eight. God saw that I was getting tired, so He sent them. They help me tidy up my room when my things are scattered. I have a lot of work, and they assist me. We help each other wash the dishes and tidy up the house together.“ The next sub-theme is academic assistance, where children described it as collaborative studying, academic support, and teaching with their imaginary companions. For example, participant 4, a seven-year-old girl with a stay-at-home mother, stated, “We study together, and I become the teacher, teaching my imaginary friend the lessons.“ Participant 10, the nine-year-old girl with a stay-at-home mother, also mentioned, “When I’m not feeling well during my studies, they help me. When I have a lot of homework, they assist me in completing it quickly.“

### Assisting in adopting new schemes

Imaginary companions facilitate the process of attraction and adaptation for children through their involvement in various roles during exploratory play. The first sub-theme is animism. When children purchase new toys, they often play with them more until they discover their qualities. They attribute a sense of life to the toy and engage in interactive play with it as a living playmate. Participant 3, a seven-year-old girl with a stay-at-home mother, stated, “I became friends with it when I bought it. We talk and we’re always together. I hug it when I sleep, otherwise, I can’t fall asleep.“ Another sub-theme is character attribution (invisible companions). Cartoons, games, and dreams contain new and captivating characters that children strive to discover and imitate. Participant 19, a six-year-old boy with working parents, also mentioned, “One night I was asleep, and a baby chick came into my dream and talked to me. Since then, I became friends with it, and we play together. I’m not alone anymore.“

### Advantages of an imaginary companion

The first sub-theme was kindness exhibited by the imaginary companion. The children expressed this by using phrases such as being kind, not bullying, not complaining, and not fighting. For example, participant 16, a six-year-old girl with working parents, stated, “It’s always kind to me. My imaginary friend never bullies me and treats me with kindness.“ The second sub-theme in this section was the reduction in TV and tablet usage. Having an imaginary companion fills the child’s solitude and time, leading them to spend less time on virtual platforms and watching television, or at least reducing their usage. Participant 12, a nine-year-old girl with working parents, mentioned, “It doesn’t hurt, it just makes me watch less TV and play less on the tablet. Even my mom doesn’t know about this friend, and she says I’ve been using the tablet less lately.“ The third sub-theme was controlling the imaginary companion. The children described this by writing the life story of their constructive imaginary friend and being in control of them. Participant 33, a seven-year-old boy with working parents, said, “They don’t bother me because I control them, and I write their story.“ Participant 23, also a nine-year-old boy with working parents, admitted, “I made them myself, so I have control over them. If they bother me, I put them in my toy basket and keep them locked up.“

### Disadvantages of an imaginary companion

Sometimes, the imaginary companion can cause fear and anxiety in children. For example, participant 9, an eight-year-old girl with a stay-at-home mother, mentioned, “Some of them are very bothersome. They scare me at night with snakes and spiders.“ The second sub-theme was creating a disruptive attachment, expressed through phrases like worrying about the imaginary companion not coming back and feeling down due to the absence of the imaginary companion. Participant 13, a nine-year-old girl with working parents, said, “I get worried when I don’t see them. I keep thinking, what if they never come back? If they don’t come back, where will I find them?“ Participant 27, a nine-year-old boy with a stay-at-home mother, also stated, “I was bothered and depressed when they didn’t come.“ The last sub-theme in this section was the control exerted by the imaginary companion. When the imaginary companion tries to take control of the child’s normative group, it causes distress for the child. As participant 31, an eight-year-old boy with a stay-at-home mother, expressed, “It nags me and keeps telling me to do this and not do that, and it messes with my nerves.“ Participant 29, a nine-year-old boy with working parents, also mentioned, “Sometimes I get upset with it because it tells me what to do and what not to do.“ It’s important to note that while imaginary companions can bring many benefits, including fostering creativity and companionship, these experiences may vary for each child.

## Discussion

As mentioned, an imaginary companion plays an important role in the lives of only children, and the research aimed to answer the question: What is the role of an imaginary companion in the lives of only children? The findings indicated that the main roles of an imaginary companion include alleviating loneliness, engaging in play with the imaginary companion, the role of the imaginary companion in emotional regulation, conversing with the imaginary companion, guidance from the imaginary companion regarding good and bad behavior, and assistance in assimilating new scripts. These results align with the findings of Mills [33], who emphasized that Indian children, in a cultural context with limited playtime and less time for being alone, consider loneliness as an important factor in creating an imaginary companion. Majors and Baines [22] stated that children with imaginary companions were likely to be firstborn or only children, which is consistent with the findings of the researcher.

Regarding the number of close friends of children with imaginary companions, the findings align with Taylor and colleagues [34], who suggested that children who are less shy and more socially confident, preferring to establish relationships with real friends, may also have imaginary companions, and their pretend play reflects these experiences.

Additionally, the findings are consistent with the research of Gleason and colleagues [29], Majors & Baines [22], and Roberts and Blanton [35]. They stated that imaginary companions often keep children entertained and accompanied in the absence of other playmates. Therefore, the absence of a good playmate may lead a child to create an imaginary companion.

On the other hand, an imaginary companion can serve as a guide for both good and bad behavior, in line with the findings of Singer and Singer [24], who highlighted that children often create imaginary companions that embody “good” or “bad” aspects. As explained, an imaginary companion sometimes encourages a child to engage in inappropriate behavior, which aligns with the findings of Aguiar and colleagues [26] and McLewin and Muller [27], who concluded that imaginary companions may play a role in prompting children to engage in mischief, especially in more practical tasks like school assignments.

The latest role expressed by only children in this study regarding imaginary companions was that imaginary companions help attract new scenarios, which is consistent with the findings of Bouldin and Pratt [11]. They stated that imaginary companions and fantasy play in general provide ways for children to incorporate novel experiences into existing scenarios to reduce distress and foster greater positive adaptation.

Considering the findings, it can be said that some children feel lonely due to the absence of siblings, and friends, or the insufficient presence of their mothers.

The second question of the study was about the advantages and disadvantages of having an imaginary companion for only children. The findings showed that the advantages of an imaginary companion include their kindness, reduced use of television and tablets, and control exerted by the imaginary companion. The disadvantages of an imaginary companion, according to the findings, include causing fear, disrupting attachment, and controlling the child.

The control and kindness of the imaginary companion were benefits mentioned by the only children in this study, which aligns with the findings of McLewin and Muller [27] and Taylor [34]. They argued that typical and normative imaginary companions are those that remain under the child’s control and operate in ways that are beneficial to the child.

In our study, a small number of only children mentioned that their imaginary companions may harm them, scare them, make them dependent, or force them to do something, which is also in line with the findings of McLewin and Muller [27] and Silberg [10]. They stated that imaginary companions in individuals with dissociative disorders can control the child’s body, make them do things they don’t want to do, and act beyond the child’s awareness, to the extent that the child may not remember what they did.

Extensive studies show that the imaginary companion is a cross-cultural phenomenon [34]. For example, Mills [33] reported that there is no recognition of the concept of imaginary companions in India. Culturally, the type and gender of this imaginary friend may differ in different countries, but an imaginary companion exists in most cultures.

since the child creates their imaginary companion based on ideals and needs, it should certainly be beneficial and facilitative for them [35, 36] Therefore, the presence of a harmful and detrimental imaginary companion can be a sign of a disorder in children because a normal child would not create a companion that is detrimental to them, and addressing the causes and treatment of this problem should be a priority [37, 38].

## Conclusion

In general, it can be said that creating an imaginary companion is not exclusive to only children without siblings, but it is more prevalent among them, perhaps because they feel lonelier or receive more attention from their parents. By understanding and examining a child’s imaginary companion, parents can realize the roles and need that the child seeks to fulfill through the imaginary companion. Understanding the advantages of an imaginary companion allows parents to recognize the characteristics and qualities that the child appreciates in themselves, the world, and others. By understanding the disadvantages of an imaginary companion, parents can become aware of any concerns or disturbances in the child’s daily life. Therefore, exploring and understanding the concept of an imaginary companion brings us closer to the child and their world. Parents should consider the characteristics of the imaginary companion and the amount of time the child spends with it. If they notice any abnormality, excessive attachment, or disruption in the child’s daily life, they should take it seriously. Nonetheless, the nature of an imaginary companion is helpful, and if it becomes harmful and detrimental to the child’s daily life, investigating the cause and addressing the issue can be significant.

As for the limitations of the study, it should be noted that this research was only conducted on only children in non-governmental schools, and its generalizability to all children may be limited. Additionally, given that the sample included imaginative and creative children, caution should be exercised when relying on statements from younger children. Interviews with unreliable samples were excluded from the study. Therefore, it is suggested that future research be conducted on only children in both governmental and non-governmental schools. Furthermore, it is recommended to include children who have not attended preschool or kindergarten in a study to avoid the disruption or alteration of imaginary companions due to the establishment of social relationships and friendships in these settings.

## Data Availability

The datasets used and/or analyzed during the current study available from the corresponding author on reasonable request.
